# Sunglint Detection for Unmanned and Automated Platforms

**DOI:** 10.3390/s120912545

**Published:** 2012-09-13

**Authors:** Shungudzemwoyo Pascal Garaba, Jan Schulz, Marcel Robert Wernand, Oliver Zielinski

**Affiliations:** 1. Institute for Chemistry and Biology of the Marine Environment—Terramare, Carl von Ossietzky University of Oldenburg, Schleusenstraβe 1, Wilhelmshaven 26382, Germany; E-Mails: jan.schulz@uni-oldenburg.de (J.S.); oliver.zielinski@uni-oldenburg.de (O.Z.); 2. Royal Netherlands Institute for Sea Research, Physical Oceanography, Marine Optics & Remote Sensing, P.O. Box 59, Den Burg, Texel 1790AB, The Netherlands; E-Mail: marcel.wernand@nioz.nl

**Keywords:** sunglint, empirical quality control, ocean colour, coastal and shelf seas, hyperspectral sensing

## Abstract

We present an empirical quality control protocol for above-water radiometric sampling focussing on identifying sunglint situations. Using hyperspectral radiometers, measurements were taken on an automated and unmanned seaborne platform in northwest European shelf seas. In parallel, a camera system was used to capture sea surface and sky images of the investigated points. The quality control consists of meteorological flags, to mask dusk, dawn, precipitation and low light conditions, utilizing incoming solar irradiance (*E_S_*) spectra. Using 629 from a total of 3,121 spectral measurements that passed the test conditions of the meteorological flagging, a new sunglint flag was developed. To predict sunglint conspicuous in the simultaneously available sea surface images a sunglint image detection algorithm was developed and implemented. Applying this algorithm, two sets of data, one with (having too much or detectable white pixels or sunglint) and one without sunglint (having least visible/detectable white pixel or sunglint), were derived. To identify the most effective sunglint flagging criteria we evaluated the spectral characteristics of these two data sets using water leaving radiance (*L_W_*) and remote sensing reflectance (*R_RS_*). Spectral conditions satisfying ‘mean *L_W_* (700–950 nm) < 2 mW·m^−2^·nm^−1^·Sr^−1^’ or alternatively ‘minimum *R_RS_* (700–950 nm) < 0.010 Sr^−1^’, mask most measurements affected by sunglint, providing an efficient empirical flagging of sunglint in automated quality control.

## 1. Introduction

Automated and unmanned remote sensing from above-water, airborne and satellite platforms is a non-invasive approach in observing marine biochemical and geophysical characteristics on a regional or global scale [1–3]. Inevitably remote sensing measurements from these platforms are prone to meteorological conditions and sunglint contamination. Sunglint is a transient anomaly that occurs when sunlight is reflected from the seawater surface directly into the down looking optical sensor [4,5]. It is a product of Fresnel reflection from a number of ‘dancing facets’ on a wind disturbed seawater surface. Sunglint is influenced by the position of the sun, viewing angle of the optical sensor, water refractive index, cloud cover, wind direction, and speed [6–8].

Kay *et al.* [9] assessed sunglint correction models for optical measurements in marine environments identifying that most of the models rely partly on the black pixel assumption [10] and tend to some extent correct glint pixels. The black pixel assumption postulates that water leaving radiance is insignificant in the near infra-red spectrum. However, several reports have shown that in coastal and turbid waters this assumption is not valid. Hence correction models employing this assumption have a high probability of over- or underestimation of apparent and inherent optical properties [5,10–12]. Thus, development of a further sunglint flag for masking obviously contaminated measurements is desirable, hence minimising the probability of errors likely to occur when using correction models.

Wernand [13] described a meteorological quality flagging method to optimise automated hyperspectral measurements for coastal and shelf seas. It masks incoming solar irradiance (*E_S_*) taken during low light, dusk, dawn and under rainfall. Additionally, to reduce measurements affected by sunglint he suggests the use of two optical sensors looking in different azimuthal directions to measure water surface leaving radiance. The lowest water surface leaving radiance for each measurement is then assumed to have the least sunglint. While this setup is useful and minimises sunglint effects on measurements, it requires additional sensors to be installed and there is a possible risk of sunglint affecting both sensors.

In this report we aim to derive sunglint quality control flags for automated and unmanned above-water hyperspectral measurements to complement meteorological flags reported by Wernand [13]. For validation we use sky and sea surface images from a simultaneously operated camera system. The automatic sunglint detection algorithm is implemented to provide an objective way of distinguishing sunglint from non-sunglint situations.

## 2. Data and Methods

**Figure 1.**
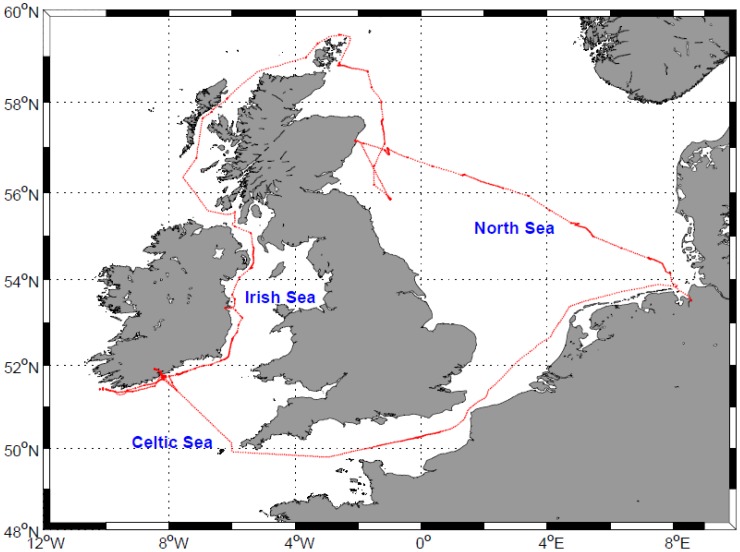
R/V Heincke HE302 cruise track (red line) where above-water hyperspectral optical measurements were taken between 21 April and 14 May 2009.

Above-water hyperspectral radiometric measurements were conducted aboard the R/V Heincke cruise HE302, between 21 April and 14 May 2009 in the northwest European shelf seas (Figure 1). The campaign was within the scope of the North Sea Coast Harmful Algal Bloom (NORCOHAB II) field campaign.

### 2.1. Instrumentation

**Figure 2.**
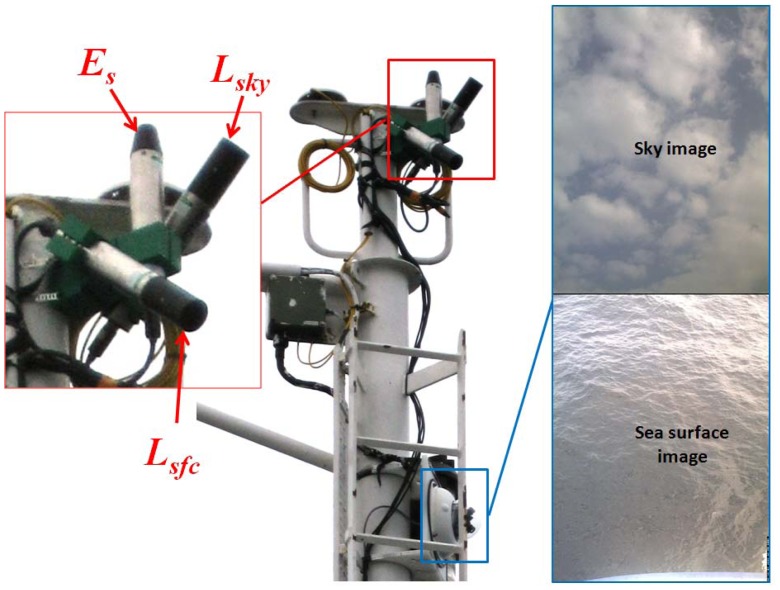
Optical sensor and camera system setup on the mast of R/V Heincke. Highlighted in red on the left side is the RAMSES hyperspectral radiometers setup, and on the right side is the DualDome D12 camera system with a sample set of captured sky and sea surface images, not to scale.

A RAMSES-ACC hyperspectral cosine irradiance meter (TriOS, Germany) was used to measure incoming solar radiation, *E_S_* (λ). Two RAMSES-ARC hyperspectral radiance meters (7° field-of-view in air), were used to detect the sea surface radiance *L_sfc_* (θ_sfc_, Φ, λ) and sky radiance *L_sky_* (θ_sky_, Φ, λ). A frame (see Figure 2) designed to hold the irradiance sensor facing upwards, with the sky and sea surface radiance sensors at zenith angles θ_sfc_ = 45° and θ_sky_ = 135°, was fixed to the mast of the ship facing starboard, 12 m above sea surface. These spectral measurements were automatically collected at 15 min intervals over a spectral range λ = 320–950 nm in steps of 5 nm.

A DualDome D12 (Mobotix AG, Langmeil, Germany) camera system with field-of-view set to 45°, was used to capture sky and sea surface images simultaneous to hyperspectral measurements, as illustrated in Figure 2. Positioning height (∼12 m above sea surface) of camera and optical sensors proved to be unaffected by sea spray. The camera's field-of-view was set congruent with the area observed by the radiometers, *L_sky_* and *L_sfc_*. Ship's position and heading were recorded by a Differential Global Position System (DGPS) and sampling times were logged in Coordinated Universal Time (UTC).

### 2.2. Methods

The first quality control step involved implementing the meteorological flagging [13] on *E_S_* (λ) measurements using MATLAB 2010a (The MathWorks, GmbH, Ismaning, Germany). The three meteorological flag conditions are:

1. *E_S_* (λ = 480 nm) > 20 mW·m^−2^·nm^−1^ setting a threshold for which significant *E_S_* (λ) can be measured,
2. *E_S_* (λ = 470 nm)/*E_S_* (λ = 680 nm) < 1 masking spectra affected by dawn/dusk radiation,
3. *E_S_* (λ = 940 nm)/*E_S_* (λ = 370 nm) < 0.25 masking spectra affected by rainfall and high humidity.

*E_S_* (λ) that passed this meteorological flagging and corresponding *L_sfc_* (θ_sfc_, Φ, λ), *L_sky_* (θ_sky_, Φ, λ) measurements were used to derive water leaving radiance, *L_W_* (θ_sfc_, Φ, λ), and remote sensing reflectance, *R_RS_* (θ, Φ, λ) according to Equation (1) [14]:

(1) $$R_{RS}=\frac{L_{W}}{E_{S}}=\frac{L_{\text{sfc}}-\left(\rho_{\text{air}-\text{sea}}\cdot L_{\text{sky}}\right)}{E_{S}}$$

where *ρ_air-sea_* is the air-sea interface reflectance factor assumed to be *ρ_air-sea_* = 0.0256 correcting for sky reflected radiation. We tested the performance of sunglint correction models by Gould *et al.* [5] Mobley [8], Lee *et al.* [4] and Ruddick *et al.* [15] using its cloud cover and wind components of Ruddick *et al.* [15] (see Supplementary Material). Optical measurements and computed parameters can be freely accessed via the PANGAEA database of the World Data Center for Marine Environmental Sciences (WDC-Mare, http://www.pangaea.de): http://www.doi.pangaea.de/10.1594/PANGAEA.759690.

#### 2.2.1. Automated Sunglint Image Detection Algorithm

**Figure 3.**
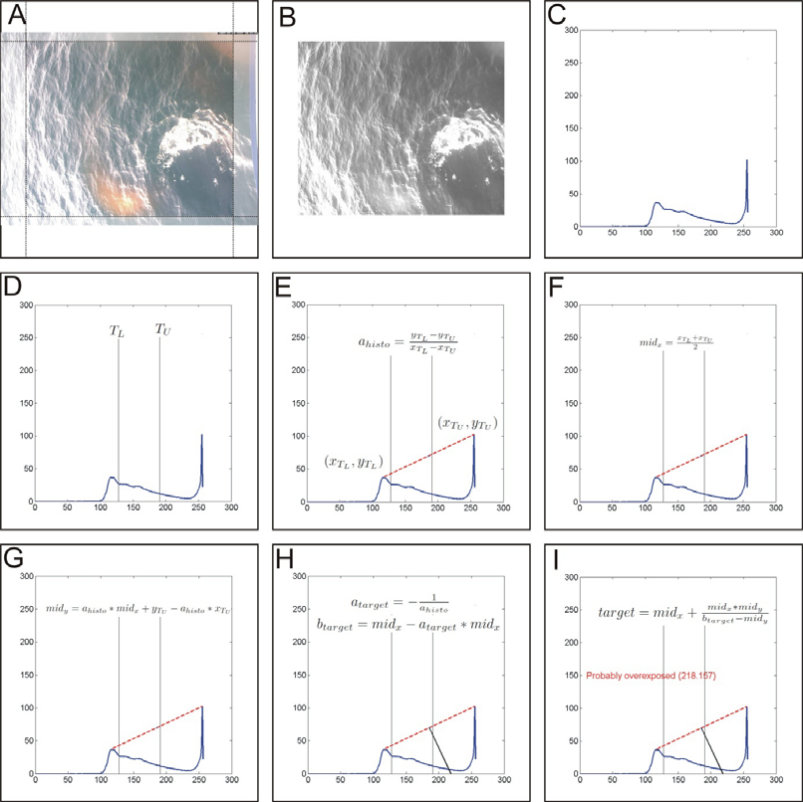
Schematic diagram of the automated sunglint image detection algorithm. (**A**) Cropping original sea surface image; (**B**) Converting cropped image to greyscale; (**C**) Extract grey level histogram of cropped and converted image; (**D**) Define lower (T_L_) and upper (T_U_) threshold; (**E**) Find position and magnitude of local maxima of darker (x_TL_, y_TL_) and brighter (x_TU_, y_TU_) colours and calculate slope of line segment; (**F**–**G**) Calculate midpoint (mid_x_, mid_y_) of line segment between (x_TL_, y_TL_) and (x_TU_, y_TU_); (**H**) Calculate slope and intercept of the target line being perpendicular to the previously calculated slope line crossing the midpoint (mid_x_, mid_y_); (**I**) Calculate target line's crossing with abscissa. If and only if this value is larger than T_U_, the image is tagged as probably overexposed.

A well exposed digital greyscale image of the sea surface normally shows several peaks scattered across the grey level histogram. Between peaks the histogram shows plateaus and has an averaged centred and balanced density distribution. When overexposed, images show a significant higher count of bright colours with white dominating. Consequently, the density distribution in the histogram shifts characteristically towards the white end where mostly one prominent peak is observed. The proposed automated sunglint detection algorithm is based on simple histogram arithmetic to evaluate the gray value ratio between dominating dark and bright counts (see Figure 3).

Processing of each full colour (24 bit colour depth) image starts with cropping image borders for 5% on all sides. As the camera is mounted stationary on the mast, field of view is prone to ship movements. Cropping has the advantage to reduce wash and white caps close to the vessel's hull, the hull itself or atmospheric interferences, depending on displacement from the vertical. Additionally it focuses the evaluated part of the image to the reading spot of the hyperspectral radiometer on the sea surface.

The cropped colour image is converted to greyscale and colour depth is reduced to *b* bits (with b < 24). In our case we use *b* = 8, resulting in a normal greyscale image with 256 levels. The histogram is computed for the greyscale image, normalised and multiplied by 2*^b^*.

Prior to determining the bright/dark ratio a lower (T_L_) and upper (T_U_) threshold is defined, with constraints 0 ≤ T_L_ < T_U_ < 2^b^. Threshold used here have been identified empirically and are in good congruence with results obtained by a human investigator. A value T_L_ = 2*^b^*/2 is a suitable first, and T_U_ = 2*^b^* × 0.75 second threshold (respectively values 128 and 192 for a 2^8^ level greyscale image). In the histogram local maxima are identified within the intervals 0 ≤ × ≤ T_L_ and T_U_ ≤ × < 2*^b^*. Values between T_L_ < × < T_U_ are excluded.

Between the two maxima a line segment is created and its slope calculated. The perpendicular to the line segment is calculated, intersecting it at the half of the length of the line segment. A special case appears if the slope of the line segment is zero, as it results in a division by zero when calculating the slope of the perpendicular. In this case a vertical line is created instead. With the previously defined constraints the slope cannot become infinity.

The intersection of the finally determined straight line with the abscissa is used as indicator for overexposure. If this value is larger than T_U_ the image is tagged as potentially overexposed.

#### 2.2.2. Spectral Analysis for Sunglint Flag

The empirical sunglint flag, based on spectral information, was developed on the premise that open seawater is assumed to absorb all light in the NIR. Thus, any light signal measured by an optical sensor would be sea surface reflectance or atmospheric scattered radiance. However, in turbid waters multiple scattering influenced by optically active seawater constituents also contributes to this light signal measurable by an optical sensor [5,12,16]. Removing a fraction *ρ_air-sea_* of the sky radiance *L_sky_* has become standard procedure to minimise sunglint in water leaving radiance *L_W_* from, above-water remote sensing. However, this approach like any correction model does not fully guarantee sunglint free measurements [4,8].

**Figure 4.**
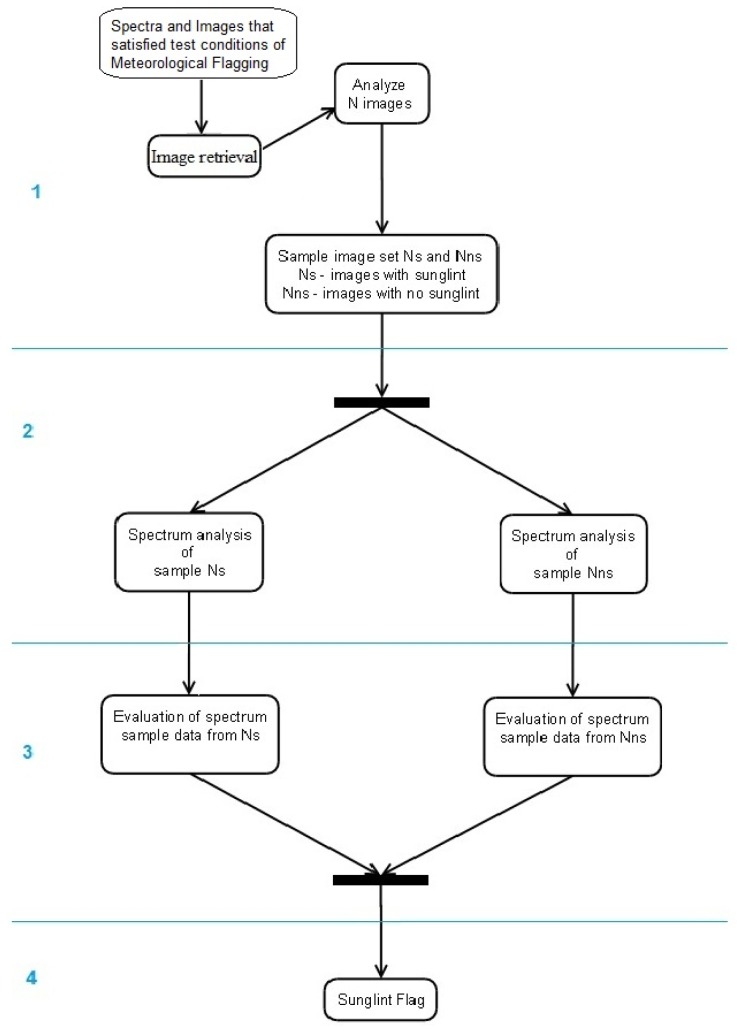
A simplified activity diagram showing the four steps involved in generating the new sunglint flag. (1) Automated sunglint image detection and sorting of spectra into *Nns*–image set without sunglint or *Ns*–sunglint-affected image set; (2) Analysis of the two spectra sets (λ = 320–950 nm); (3) Specific spectra analysis in visible λ = 320–700 nm and near infra-red λ = 700–950 nm range (4) Performance test and identification of effective sunglint flagging criteria.

Our objective is to provide a reliable sunglint flagging with the advantage of sea-surface images to validate an empirical spectral approach to eliminating measurements affected by sunglint. In Figure 4 a simplified activity diagram illustrates the steps that were implemented in this sunglint flag investigation and evaluation:

1. The automated sunglint image detection algorithm (Section 2.2.1) was applied to sea surface images that are matching the unmasked spectra validated with the meteorological flagging [13]. Based on the computed probability of sunglint contamination in the images, they were classified into *Nns*–image set without sunglint or *Ns*–sunglint-affected image set.
2. Spectra analysis of the two sets *Nns* and *Ns* was used to identify unique characteristics with respect to *L_W_* (λ) and *R_RS_* (λ). This included the investigation of each individual spectra and mean spectra for the two sets over the whole spectrum range (λ = 320–950 nm), to obtain a general overview on typical spectra for the sets *Nns* and *Ns*;
3. To obtain distinguishing features for spectra data sets in *Ns* and *Nns* utilising findings from step 2 (*i.e.*, spectrum shape, behaviour and magnitude variations of both *L_W_* (λ) and *R_RS_* (λ) in the VIS and NIR) a combination of inequality equations and band ratios was implemented. These combinations include:
  - using the NIR mean spectra to obtain threshold values unique in set *Nns* and *Ns*. This procedure was repeated using the NIR minimum spectra obtained from the statistical analysis of the *Nns* and *Ns* sets respectively,
  - performing spectral band ratioing on characteristic spectral bands both in VIS and NIR, here λ = 400 nm, 460 nm, 760 nm, 940 nm [9,13,17].
4. To test the performance of the above mentioned procedures a reanalysis of the original united data set of all valid spectra from step 1 was run. Based on the comparison of this computation with the independent image algorithm (Section 2.2.1.) effective sunglint flagging criteria were identified.

## 3. Results and Discussion

A total of 629 from 3,121 spectral measurements passed the test conditions of the meteorological flagging. The image assessment of these unmasked 629 spectra came up with 501 images free of sunglint, *Nns (having least visible/detectable white pixel or sunglint)*, and 128 sunglint-affected images, *Ns (having too much or detectable white pixels or sunglint)*.

### 3.1. Sunglint Image Analysis

**Figure 5.**
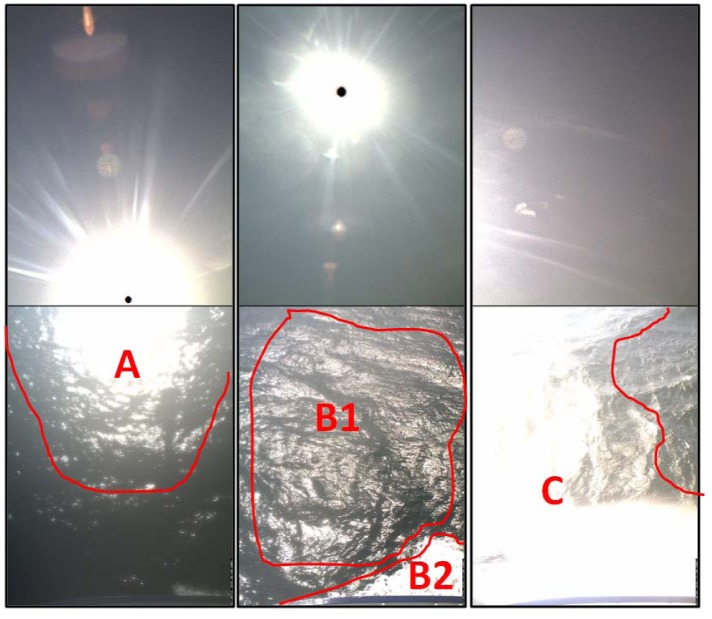
Starboard side sky (**top**) and sea surface (**bottom**) images captured during R/V Heincke field campaign HE302 showing sources of erroneous measurements; (A)—sunglint; (B1)—sunglint and (B2)—whitecap or foam; and (C)—a combination of sunglint, whitecaps and foam.

Automated and unmanned optical measurements from a seaborne platform are challenging. It is difficult to adhere to recommended sensor setups for θ_sfc_, θ_sky_, Φ; thus it is inevitable to collect measurements affected by sunglint, whitecap and foam [18–20]. Figure 5 demonstrates typical situations also noted during the image inspection in step 2 [Section 2.2.1 and Figure 3(a,b)].

Using the automated sunglint image detection algorithm, the output was either an image with a probability of sunglint or without. Sunglint (Figure 5(A,B1,C)) as well as whitecaps and foam (Figure 5(B2,C)) were identified as main sources of contamination, with the latter two presumably resulting in similar sunlight influenced spectral patterns. This image algorithm detects overexposure which is a result of sunglint and/or whitecaps and foam; we however assume that sunglint plays a major role.

### 3.2. Sunglint Flag

**Figure 6.**
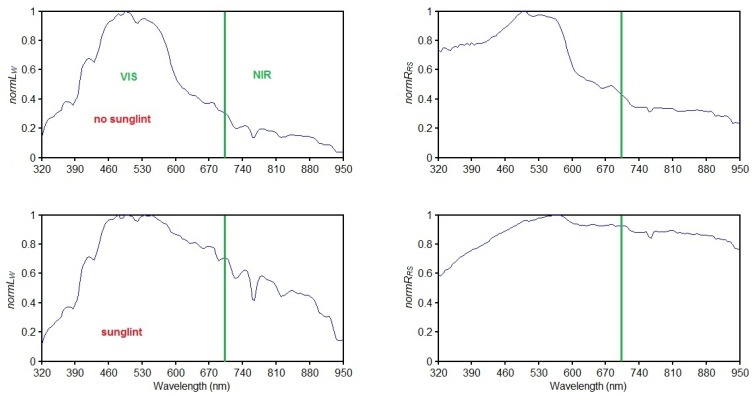
Normalised mean spectral shapes for the sunglint free set *Nns* (**top**) and sunglint set *Ns* (**bottom**). The green line highlights the spectral limits for the VIS (λ = 320–700 nm) and NIR (λ = 700–950 nm).

A spectral assessment of the sample sets indicated that *R_RS_* (NIR) and *L_W_* (NIR) are significantly enhanced for *Ns*, compared to *Nns*. The enhanced spectra in set *Ns* were assumed to be a result of sunglint, whitecaps, and foam. In Figure 6 normalised mean spectral shapes for *Nns* (501 spectra) and *Ns* (128 spectra) illustrate these findings. Data normalisation was applied to simplify the visual comparison of spectra, dividing each *L_W_* (λ) and *R_RS_* (λ) measurement by the maximum value for each measurement. However, for determining the flagging criteria, the actual computed spectral measurements *L_W_* (λ) and *R_RS_* (λ) were used.

**Figure 7.**
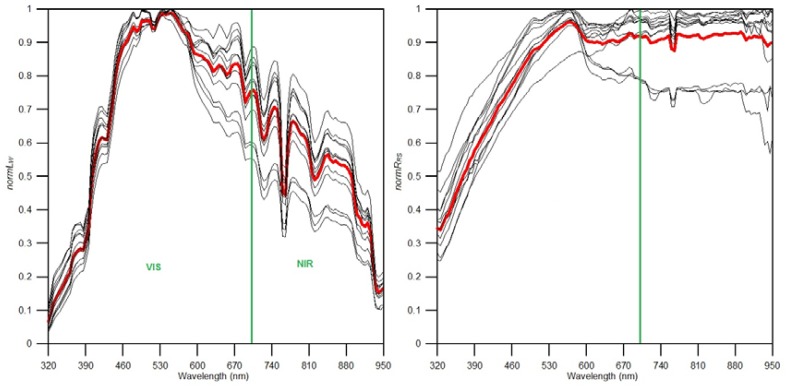
Thirteen samples of spectral shapes for sea surface images strongly affected by sunglint from the sunglint image set *Ns*. On the left is the normalized water leaving radiance, *normL_W_* and on the right is the normalized remote sensing reflectance, *normR_RS_*. It is observed that sunglint enhances the measured signal especially in the NIR for both water leaving and remote sensing reflectance. The red spectrum shows the mean spectral shape and the green line highlights the VIS and NIR spectral ranges.

Figure 6 shows how the mean spectra, with respect to *normL_W_* and *normR_RS_* values, are enhanced in the presence of sunglint in both the VIS and NIR compared to the non-sunglint situation. To further investigate sunglint spectral characteristics, 13 of the 128 sea surface images in *Ns* were identified to be highly affected by sunglint (see Figure 7). Their spectral shapes also reveal the same trend of enhanced water leaving and reflectance signal over the measured spectrum.

Spectral band ratios, a conventional approach in remote sensing algorithms, were implemented to identify differences in *Nns* and *Ns*. In previous reports related to sunglint correction, the following characteristic spectral bands were used mainly due to their physical properties: (a) oxygen absorption band (λ ≈ 760 nm) which has been used in a prior sunglint correction model [21]; (b) water and precipitable water vapour absorption band (λ ≈ 940 nm) known to interact with solar radiation in the NIR [17,22]; and (c) coloured dissolved organic matter absorption from UV to visible, here investigated at (λ ≈ 400 nm) and (λ ≈ 460 nm), contributing to sunglint through multiple scattering [23,24].

Several tests were implemented to obtain the most effective sunglint flagging. Test results are summarized in Table 1. A ranking was introduced grouping the methods according to their performance in the detection of sunglint affected data.

The method of thresholds was chosen because it is a widely used technique in developing flagging and validation algorithms [13,25,26]. Another benefit of using a threshold is the possibility of adjusting them to improve sensitivity of algorithms or models [27]. The threshold values were obtained by repetitive testing of possible threshold values *i.e.*, after statistically computing the min, max and mean for spectra in the NIR. The computed statistics provided a starting test point and altered to obtain the best threshold values aimed at; (i) masking/eliminating as many measurements in the sunglint-affected set *Ns*; and (ii) unmasking/keeping as many measurements in the sunglint free set *Nns*. The performance test summarized in Table 1, revealed the most effective sunglint flagging criteria if the effectiveness of both tasks is taken into account;

- ‘mean (*L_W_*)_NIR_ < 2 mW·m^−2^·nm^−1^·Sr^−1^’, being 97% effective in sets *Nns* and 91% effective in *Ns* (91%); Or alternatively
- ‘minimum (*R_RS_*)_NIR_ < 0.010 Sr^−1^’, with an effectivene*ss* of 95% in sets *Nns* and 94% in *Ns*.

### 3.3. Remote Sensing Reflectance

In the previous section, a sunglint flagging was identified which can be implemented using either *L_W_* or *R_RS_*. Both, the meteorological flagging conditions [13] and herein proposed sunglint flag criteria are presented in Table 2. The first three flags rely on the incoming radiation, *E_s_*, thus masking measurements taken during dusk or with too low incoming solar radiation (Flag 1), during dawn (Flag 2), or under rainfall (Flag 3). Equation 1 is then applied to derive the water leaving radiance, *L_W_* and remote sensing reflectance, *R_RS_*, followed by the sunglint flag validation. In this study the sunglint flag was implemented using *L_W_* (Flag 4a) because it is the first product of Equation 1 but can be replaced by *R_RS_* (Flag 4b) as summarised in Table 2. In order not to limit flagging to one remote sensing product, e.g., *L_W_* or *R_RS_* only, Flag 4a or Flag 4b were proposed.

## 4. Conclusions and Outlook

In this study we developed two alternative sunglint flagging criteria as a quality control procedure for unmanned and automated above-water platforms measuring water reflectance. To provide an independent evaluation method for sunglint situations, an automated sunglint image detection algorithm was successfully implemented. The Flag 1 (the minimal *E_S_* accepted) is a bit arbitrary and certainly depends on the geographical position. Flag 2 can be discussed, although this flag does not depend on the region but more or less on sun rise and fall. We choose for a more or less average shaped spectrum (comparable with the form of the Es spectrum at high noon, only amplitude differs. Flag 4 is depends on the region as we are using a empirical approach and therefore makes the use of the camera important.

We assumed sunglint to be the main cause of error in the collected measurements after applying the meteorological flagging [13]. However, the influence of whitecaps and foam has been reported to cause both: (i) a decrease in reflectance in the NIR due to radiation absorption by large air bubbles [28,29], or physical coolness of residual foam [30]; (ii) enhanced reflectance occurring as soon as waves break generating thick strong reflecting foam [20]. The image analysis revealed that sunglint was also present in sea surface images influenced by whitecaps/foam, and it was not possible to distinguish how each of them contributes to the spectra. It was therefore assumed that for the available measurements sunglint and whitecaps/foam led to contaminated measurements. It is for this reason that in future studies the contribution of whitecaps and foam be specifically investigated with respect to sunglint flagging, possibly utilising novel glint measurement apparatus e.g., [31,32] integrated with sunglint and wave models such as [4,5,7,15,33].

The sunglint image detection algorithm introduced here has shown to be a valuable additional tool for detecting contaminated sensor readings. However, the used values for T_L_ and T_U_ are empirical. Results with the given values have shown a good congruence with visual inspection by a human investigator. It might useful to adjust thresholds to specific situations or locations. Higher counts in the bright part of the histogram result in the situation that the ‘target’ line intersects the x-axis at higher values. Thus, increased numbers of bright pixels simultaneously emphasize the shape of the histogram on the ‘bright side’. Using different values for T_L_ and T_U_ impacts the sensitivity of the function for darker/brighter pixels.With respects to sensor setup we suggest that prior to automated and unmanned optical measurements the Solar Position Algorithm [34] be utilised to predict or identify optimal relative angle of optical sensor setup to the sun's position despite the limiting factors of R/V pitch, roll and yaw motions [14,18,35]. Here the idea is not to necessarily change the cruise track but to perform underway optical measurements avoiding sunglint. Alternative approaches to minimise sunglint, but increasing technical requirements, would be (a) to turn the sensors by some automatic device e.g., RFlex (http://www.sourceforge.net/p/rflex/wiki/Home/) or (b) to have more sensors and cameras looking at different azimuthal directions. However for fixed platforms, such as piles, these alternative approaches can be avoided if SPA is utilised beforehand to limit sunglint influenced measurements. Furthermore, we provide a set of step that can be followed to perform sunglint correction on above-water automated measuring platforms (Appendix A).

Recently operational oceanographic observatories are becoming more prominent and at the same time hyperspectral radiance sensor technology becomes increasingly affordable [2,3]. Therefore the application of reflectance measurements above the water surface, from stationary and moving platforms alike, is expected to significantly grow in numbers. Given this enormous amount of data, favourably processed in real-time, effective quality control procedures like the ones discussed here are more than supporting tools, they are a crucial prerequisite for trustworthy and manageable information.

## Figures and Tables

**Table 1.** A summarized evaluation of the most effective sunglint flagging ranked according to their performance in masking sunglint spectra in a reanalysis. To check for effectiveness, the percentage E (*Ns*) % and E (*Nns*) % was derived by dividing the number of spectra masked or unmasked by each condition with the actually number of spectra in sets (sunglint set *Ns*—128 spectra and non sunglint set *Nns*—501 spectra).

**Sunglint Flag Test Condition**	**Sunglint Affected Observations Masked**	**E (*Ns*) %**	**Sunglint Free Observations Unmasked**	**E (*Nns*) %**
Minimum (*L_W_*)_NIR_ < 0.3 mW/m^2^·nm·Sr	123	96	454	91
Mean (*R_RS_*)_NIR_ < 0.010 Sr^−1^	123	96	453	90
Minimum (*R_RS_*)_NIR_ < 0.010 Sr^−1^	121	95	471	94
Minimum (*L_W_*)_NIR_ < 0.4 mW/m^2^·nm·Sr	119	93	478	95
Mean (*L_W_*)_NIR_ < 2.000 mW/m^2^·nm·Sr	117	91	487	97
Minimum (*L_W_*)_NIR_ < 0.5 mW/m^2^·nm·Sr	115	90	486	97
Minimum (*R_RS_*)_NIR_ < 0.012 Sr^−1^	115	90	479	96
Mean (*R_RS_*)_NIR_ < 0.015 Sr^−1^	112	88	475	95
*L_W_* (940)/*L_W_* (400) < 0.145	111	87	480	96
Mean (*L_W_*)_NIR_ < 2.500 mW/m^2^·nm·Sr	110	86	492	98
*L_W_* (765)/*L_W_* (460) < 0.290	110	86	477	95
Minimum(*R_RS_*)_NIR_ < 0.015 Sr^−1^	109	85	490	98
*R_RS_* (765)/*R_RS_* (400) < 0.700	109	85	468	93
*R_RS_* (760)/*R_RS_* (400) < 0.700	108	84	470	94
*L_W_* (940)/*L_W_* (460) < 0.090	108	84	488	97
*R_RS_* (760)/*R_RS_* (460) < 0.650	107	84	473	94

**Table 2.** Summarised meteorological and sunglint flag conditions. The meteorological criteria [13] are represented by Flags 1–3. The sunglint flag can be implemented either as Flag 4a or Flag 4b depending on availability of measured water leaving radiance, *L_W_* or remote sensing reflectance, *R_RS_*.

**Flag Name**	**Purpose**	**Test Conditions**
Flag 1	The ‘minimal flag’ sets the lower limit for which significant incoming solar radiation can be measured.	*E_s_* (480 nm) > 20 mW/m^2^ nm
Flag 2	The ‘shape flag’ will mask optical measurements influenced by dusk ‘red colouring of the sky’ or dawn radiation.	*E_s_* (470 nm)/*E_s_* (680 nm) < 1
Flag 3	The ‘rainfall flag’ will mask optical measurements influenced by precipitation or high humidity.	*E_s_* (940 nm)/*E_s_* (370 nm) > 0.25
Flag 4a	The ‘sunglint flag’ will mask optical measurements influence by sunglint based on L_W_.	Mean L_w_ (700–950 nm) < 2 m W/m^2^ nm Sr
Flag 4b	Alternative ‘sunglint flag’ based on R_RS_.	Minimum R_RS_ (700–950 nm) < 0.010 Sr^−1^

## Appendix A

The following recommendations assume automated and unmanned platform above-water radiometry is performed using the setup explained in this report. For a detailed explanation of the correction models refer to the respective protocols.

Prerequisite information:

- Wind speed [m/s]
- Cloud cover
  ○ L_sky_(750)/E_s_(750) [15]
  ■ <0.05 means clear sky
  ■ ≥0.05 means cloudy sky
- Ship heading
- Ship GPS data
- Sea surface images

### Step 1

Apply three meteorological flags:

1. *E_S_* (λ = 480 nm) > 20 mW·m^−2^·nm^−1^ setting a threshold for which significant *E_S_* (λ) can be measured.
2. *E_S_* (λ = 470 nm)/*E_S_* (λ = 680 nm) < 1 masking spectra affected by dawn/dusk radiation.
3. *E_S_* (λ = 940 nm)/*E_S_* (λ = 370 nm) < 0.25 masking spectra affected by rainfall and high humidity [13].

### Step 2

#### Alternative 1

Usage of the collected sea surface images and implementation of the sunglint detection algorithm. It eliminates images too contaminated with sunglint. Respective matching spectra of images successfully passing the image detection algorithm are used in Step 3.

#### Alternative 2

Determine L_W_ and R_RS_ and apply the spectral conditions supplied for sunglint flagging. To validate and verify flagging performance cross check with the image algorithm.

If not satisfactory, follow the procedures described in Section 2.2.2. ‘Spectral Analysis for Sunglint Flag’ and adjust the sunglint flag conditions appropriately.

#### Alternative 3

Determine the relative azimuthal angle of the sensor to the sun using e.g., Solar Position Algorithm, SPA [34]. Using the ship's position the SPA will compute the sun's azimuthal and zenith angle at a given space-time spot.

Extract spectra collected in the optimal relative azimuthal angle of sensor to sun 90° ≤ Φ ≤ 135°.

In this case if required, verify measurements with the image sunglint detection algorithm to further eliminate images with too much glint or above average white pixels.

Proceed with next step.

### Step 3—Sunglint Correction

To determine which coefficient optimally removes sea surface reflectance/sunglint a number of methods are available. It is important to test them and decide on which correction model to apply from these:

#### Lee *et al.* [4]

Lee *et al.*, recommends a spectral optimization where *ρ_air-sea_* = 0.022 = Fresnel Reflectance. Additionally a bias delta (Δ), obtained from comparing modeled *R_RS_* and in-situ *R_RS_*, is then used as residual sea surface reflectance. To calculate *R_RS_* use Equation (2):

(2) $$R_{RS}=\frac{L_{\text{sfc}}}{E_{S}}-\frac{\rho_{\text{air}-\text{sea}}\cdot L_{\text{sky}}}{E_{S}}-\Delta$$

#### Ruddick *et al.* [15]

Ruddick *et al.*, provides a model based on cloud cover and wind speed, W that is aimed at correcting sunglint for measurements from turbid to very turbid water, to calculate *R_RS_* use Equation (3).

- L_sky_(750)/E_d_(750) test for presence of cloud cover in L_sky_ field of view [15]
  ○ <0.05 means clear sky *ρ_air-sea_* = 0.0256 + 0.00039 W + 0.000034 W^2^
  ○ ≥0.05, cloudy sky *ρ_air-sea_* = 0.0256
    (3) $$R_{RS}=\frac{L_{W}}{E_{S}}=\frac{L_{\text{sfc}}-\left(\rho_{\text{air}-\text{sea}}\cdot L_{\text{sky}}\right)}{E_{S}}$$

#### Gould *et al.* [5]

According to Gould *et al.* [5] correction can be performed for sunglint by assuming that sunglint consists of (a) spectrally variable sky radiance *ρ_air-sea_* (0.021) × L_sky_ and (b) spectrally-flat sunglint and cloud reflected radiance, B. To calculate *R_RS_* use Equation (4):

(4) $$R_{RS}=\frac{L_{\text{sfc}}}{E_{S}}-\frac{\rho_{\text{air}-\text{sea}}\cdot L_{\text{sky}}}{E_{S}}-B$$

#### Mobley [8]

Mobley provides a number of simulations and recommends *ρ_air-sea_* = 0.028 for clear skies and wind speed < 5 m/s. For higher wind speeds change adjust *ρ_air-sea_* accordingly. For cloudy skies at all wind speeds he recommends *ρ_air-sea_* = 0.028. However, take note that the higher the *ρ_air-sea_* is you might over correct and get negative reflectance in the NIR. To calculate *R_RS_* use Equation (5):

(5) $$R_{RS}=\frac{L_{W}}{E_{S}}=\frac{L_{\text{sfc}}-\left(\rho_{\text{air}-\text{sea}}\cdot L_{\text{sky}}\right)}{E_{S}}$$
